# Hyperbaric oxygen therapy and Fournier’s gangrene: a systematic review and meta-analysis

**DOI:** 10.4103/mgr.MEDGASRES-D-25-00227

**Published:** 2026-01-23

**Authors:** Anish Patel, Deepak Batura

**Affiliations:** Department of Urology, London North West University Healthcare NHS Trust, London, UK

**Keywords:** debridement, Fournier’s gangrene, hyperbaric oxygen, hyperbaric oxygen therapy, hospital length of stay, meta-analysis, mortality, necrotizing fasciitis, sepsis, systematic review

## Abstract

**Facts**

1. A meta-analysis demonstrated hyperbaric oxygen therapy reduces the relative risk of death in patients suffering from Fournier’s gangrene.
2. The present study has revealed no statistically significant differences in terms of hospital length of stay or the number of surgical debridements in patients with Fournier’s gangrene between the hyperbaric oxygen therapy and control groups.
3. The extant evidence supports the use of hyperbaric oxygen therapy as an ancillary measure of Fournier’s gangrene following prompt surgical debridement and antimicrobial therapy, rather than as a primary treatment modality.

**Open Questions**

1. What are the optimal hyperbaric oxygen treatment protocols (pressure, duration, number of sessions) for patients with Fournier’s gangrene?
2. Which patient subgroups (e.g. comorbidities, infection severity) derive the most benefit from hyperbaric oxygen therapy?
3. How does hyperbaric oxygen therapy affect resource utilization and cost-effectiveness, particularly in healthcare systems with limited hyperbaric infrastructure?

Fournier’s gangrene is a rare, rapidly progressive necrotizing soft tissue infection of the genitals and perineum that continues to carry a high mortality rate. Hyperbaric oxygen therapy has been proposed as an adjunctive treatment, but its efficacy remains uncertain. We conducted a systematic review and meta-analysis of studies comparing hyperbaric oxygen therapy with standard care in adults with Fournier’s gangrene. A comprehensive search of PubMed/MEDLINE, Embase, Cochrane Library, Scopus, and Web of Science identified 13 retrospective studies including 322 patients treated with hyperbaric oxygen therapy and 366 controls. Hyperbaric oxygen therapy was associated with a significantly lower mortality rate compared with control groups. Secondary outcomes, including hospital length of stay and number of debridements, showed inconsistent results and did not differ significantly between groups. These findings suggest that hyperbaric oxygen therapy may reduce mortality in patients with Fournier’s gangrene and could be considered as an adjunctive therapy.

## Introduction

Necrotizing fasciitis is a rare but often life-threatening bacterial infection of soft tissues, characterized by rapid necrosis of tissues, which requires urgent medical and surgical attention.^1^ French dermatologist, Jean Alfred Fournier,^2^ first published in 1883, a case series of five patients presenting with necrotizing fasciitis originating from the scrotum. Given that the men were previously healthy, the underlying cause was unknown at the time. Thus, it was assumed to be an idiopathic disease.^3^ Today, Fournier’s gangrene is defined as necrotizing fasciitis of the scrotal, perineal, anal and genital regions.^4^ Diagnosis can be challenging and often missed due to the varied severity of presentation as well as the condition’s propensity to involve deeper tissues and fascia while sparing the overlying skin and superficial tissue in the early stages.^5^ Fournier’s gangrene is typically a polymicrobial infection that spreads rapidly along fascial planes.^6^ This leads to severe inflammation, obliterative endarteritis, vessel thrombosis, tissue ischemia & necrosis.^7^ Without prompt intervention, a localized infection can escalate within hours, progressing to sepsis, septic shock, multiorgan dysfunction and ultimately death.^8^

Management of these patients is complex and necessitates a multi-disciplinary approach. However, there is broad consensus on the initial steps of management; antibiotic therapy covering Gram-positive, Gram-negative and anaerobic organisms, along with urgent and aggressive surgical debridement of necrotic tissue.^9^ Depending on the disease severity, multiple debridements may be required, with reconstructive surgery often required at a later stage.^10^ Despite this, however, global mortality of Fournier’s gangrene has remained high, historically reported between 20–40%.^11^,^12^ Adjunctive therapies, therefore, could play a significant role in improving mortality and morbidity, and research is clearly warranted.

One such adjunct is hyperbaric oxygen therapy, first documented in 1662, when British physician Henshaw placed patients in a pressurized air chamber.^13^ Since then, the therapy has evolved considerably, with modern therapy involving inhalation of 100% oxygen in a controlled pressurized environment (usually at 1.5–3 atm [1 atm = 101.325 kPa]).^14^ Inhalation of 100% oxygen creates a diffusion gradient that enhances oxygen delivery from hyper-oxygenated lungs to hypoxic tissues (hyperoxia) and the increased pressure raises the blood oxygen concentration (hyperoxemia).^15^ Hyperbaric oxygen therapy currently has a wide variety of clinical applications, namely, but not limited to: gas embolism,^16^ thermal burn injury,^17^ carbon monoxide poisoning,^18^ decompression sickness,^19^ severe anemia,^20^ and delayed radiation injury.^21^ In the context of necrotizing soft tissue infections, hyperbaric oxygen therapy has been proposed to (i) enhance tissue oxygenation, (ii) exert direct bactericidal effects on anaerobic organisms (Clostridium perfringens growth is restricted at O_2_ tensions up to 70 mmHg), (iii) reduce bacterial toxin production (*Clostridium perfringens* alpha-toxin production is stopped at tensions of 250 mmHg,^22^ (iv) activate T-helper cells, and (v) promote angiogenesis and collagen synthesis.^23^ However, consensus on its use in Fournier’s gangrene remains divided, with varying recommendations across clinical settings.^24^ Previous reviews of hyperbaric oxygen therapy have largely focused on necrotizing soft tissue infections in general.^25^ In contrast, this review is Fournier’s gangrene–specific, examines mortality as the primary outcome and adopts a broader inclusion criterion to address the scarcity of available evidence. This approach provides a more targeted and clinically relevant assessment of hyperbaric oxygen therapy’s potential survival benefit in this rare, high-risk condition.^26^

## Methods

This systematic review was conducted in accordance with the Preferred Reporting Items for Systematic Reviews and Meta-Analyses (PRISMA) guidelines.^26^ The aim was to assess the efficacy and impact of Hyperbaric Oxygen therapy on clinical outcomes in patients with Fournier’s gangrene.

### Search strategy

A comprehensive literature search was conducted across the following electronic databases: PubMed/MEDLINE, Embase, Cochrane Library, Scopus, and Web of Science. The search strategy included combinations of Medical Subject Headings (MeSH) and keywords including: “Fournier’s gangrene,” “necrotizing fasciitis,” “hyperbaric oxygen therapy,” “HBOT,” “high pressure oxygen,” “negative pressure wound healing,” and “medical gases.” Boolean operators (AND, OR) were used to optimize search sensitivity. Reference lists of relevant articles and reviews were also manually screened for additional eligible studies. Studies published within the last 20 years were included. Only studies published in the English language were included.

### Eligibility criteria

Studies were included in this review if they examined adult patients (aged 18 years or older) diagnosed with Fournier’s gangrene. The intervention of interest was hyperbaric oxygen therapy administered as an adjunct to standard care, which typically comprises resuscitation, broad-spectrum antibiotic therapy, and surgical debridement. Eligible studies included those that compared outcomes between patients receiving adjunctive hyperbaric oxygen therapy in addition to standard care and those receiving standard care alone. The primary outcome of interest was mortality, and studies were required to report on this outcome to be eligible for inclusion. Secondary outcomes included hospital length of stay and the number of surgical debridements. Although these secondary outcomes were not mandatory for inclusion, they were extracted and recorded when reported. A range of study designs was considered, including randomized controlled trials, cohort studies, case-control studies, and case series involving five or more patients. Only studies published in English were included. Studies were excluded if they involved patients with necrotizing soft tissue infections other than Fournier’s gangrene. Additionally, studies were excluded if they were conference abstracts, case series involving fewer than five patients, single case reports, review articles, editorials, or animal studies. Studies that did not report data relevant to the efficacy of hyperbaric oxygen therapy were also excluded from the review.

**Figure 1:**
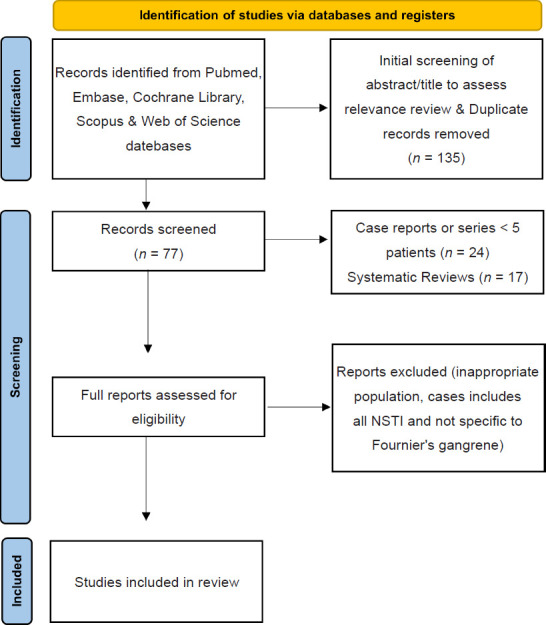
PRISMA flow chart demonstrating study selection and eligibility screening. NSTI: Necrotizing soft tissue infection; PRISMA: Preferred Reporting Items for Systematic Reviews and Meta-Analyses.

A total of 212 studies were identified through the initial database search. After reviewing the title/abstract for relevance and removing duplicates, 77 studies were screened. Of these, 42 studies were excluded due to study design, and 22 studies were excluded as they were not appropriate for our review. Given the lack of high-quality studies available for our study protocol, four studies were included that had no direct control arm for comparison. Mortality rates from these studies would be compared to historical mortality rates for Fournier’s gangrene with no adjunctive therapy; for the purpose of this study, it is accepted at 25%.^11^ In total, 13 studies met the inclusion criteria and were included in the final analysis (**Figure 1**).

### Data extraction

Data extraction was performed independently by the two authors (AP & DB) using a standardized extraction template. Any discrepancies were discussed and resolved by consensus. Study characteristics (author, year, country, design, and number of centers), sample sizes and demographic details (age, and sex), and clinical outcomes (mortality rates, hospitalization time, and number of debridements) were extracted (**Table 1**).

**Table 1:** A summary of included studies including primary and secondary clinical outcomes

Author	Year	Study design	Group	Sample size	Deaths	Mortality (%)	Hospitalization time (d)	Number of debridements
Michalczyk et al.^27^	2022	Retrospective cohort	HBOT	13	0	0.0	26	–
			Control (No-HBOT)	22	4	18.2	23	–
Tutino et al.^28^	2022	Retrospective cohort	HBOT	12	1	8.3	21.6	–
			Control (No-HBOT)	11	1	9.1	28	–
Feres et al.^29^	2021	Retrospective cohort	HBOT	79	2	2.5	–	–
			Control (No-HBOT)	118	34	28.8	–	–
Grabińska et al.^30^	2021	Retrospective cohort	HBOT	8	0	0.0	29	2
			Control (No-HBOT)	5	0	0.0	51	3
Creta et al.^31^	2020	Retrospective cohort	HBOT	72	14	19.4	–	–
			Control (No-HBOT)	89	32	36.0	–	–
Anheuser et al.^32^	2018	Retrospective cohort	HBOT	17	0	0.0	40.3	13.3
			Control (No-HBOT)	45	2	4.4	22.5	4.8
Hung et al.^33^	2015	Retrospective cohort	HBOT	12	0	0.0	–	–
			Control (No-HBOT)	48	16	33.3	–	3.1
Li et al.^34^	2015	Retrospective cohort	HBOT	16	2	12.5	31.4	1.32
			Control (No-HBOT)	12	4	33.3	31.25	2.17
Mindrup et al.^35^	2005	Retrospective cohort	HBOT	26	7	26.9	21	1
			Control (No-HBOT)	16	2	12.5	25	1
Rosa and Guerreiro^36^	2015	Case series	HBOT (all)	24	5	20.8	–	–
Inácio et al.^37^	2019	Case series	HBOT (all)	23	5	21.7	20.13	2.61
Gülşen et al.^38^	2019	Case series	HBOT (all)	14	1	7.1	–	–
Milanese et al.^39^	2015	Case series	HBOT (all)	6	0	0.0	–	1.17

–: Not reported; HBOT: hyperbaric oxygen therapy.

### Data synthesis

A quantitative synthesis was performed across the 13 included studies, consisting of nine retrospective cohort studies and four case series. Initially, a descriptive comparison of individual studies was conducted, focusing on the primary outcome (mortality) and secondary outcomes (length of hospitalization and number of debridements). Study characteristics, patient populations, and reported outcomes were reviewed and summarized to identify patterns and variations across studies (**Table 1**).^27^,^28^,^29^,^30^,^31^,^32^,^33^,^34^,^35^,^36^,^37^,^38^,^39^

Following this, outcomes were grouped and averaged to allow for descriptive comparison between patients treated with hyperbaric oxygen therapy and those who did not receive hyperbaric oxygen therapy (**Table 2**). For studies that did not include a non-hyperbaric oxygen therapy control group (i.e., case series), direct comparison within the study was not possible. To enable interpretation of mortality outcomes, the observed mortality rates in these studies were compared against a literature-reported average mortality rate of 25%^11^ in Fournier’s gangrene. Subgroup analyses were not performed due to inconsistent or incomplete reporting of patient characteristics. In several studies, such data were either unavailable or only reported for the total population, precluding stratified analysis. Furthermore, this was beyond the scope of our primary objectives but could be considered in future reviews.

**Table 2:** Grouped summary of primary & secondary study outcomes in Fournier’s gangrene patients for HBOT and control groups

Group	*n*	Deaths	Mortality rate (%)	Median hospitalisation time (d)	Median number of debridements
HBOT	322	37	11.5	26.0	1.7
Control (No-HBOT)	366	95	26.0	28.0	2.6

HBOT: Hyperbaric oxygen therapy.

Where data permitted, a meta-analysis was performed. The meta-analysis for mortality included nine retrospective cohort studies that provided clearly defined hyperbaric oxygen therapy and non-hyperbaric oxygen therapy groups. For secondary outcomes, meta-analyses could only be conducted using three studies that reported the outcome of interest and had two clearly defined treatment groups.

### Statistical analysis

Meta-analyses were conducted to give a single estimate of the group differences, using a random-effects model employing the Der Simonian and Laird method, regardless of the amount of heterogeneity between studies. Differences between groups for individual studies, and for all studies combined, were quantified using relative risks for binary outcome measures, and by the mean difference between groups for continuous outcomes. Heterogeneity was assessed using the Chi-square test and the *I*² statistic (which quantifies the percentage of the variability in effect estimates that is due to heterogeneity rather than sampling error), with substantial heterogeneity defined as an *I*^2^ value greater than 50% or a statistically significant Chi-square test result.

### Bias assessment

Two authors independently scored each study using the Newcastle-Ottawa Scale for observational studies.^40^ A total score of 9 can be given after assessing the method of selection, comparability and outcomes. Scores of 7–9 suggest high quality, 4–6 fair quality and 0–3 poor quality.

## Results

### Study characteristics

All 13 included studies were retrospective; no randomized control trials were identified during our literature search comparing hyperbaric oxygen therapy with standard care for Fournier’s gangrene. The studies were conducted across a range of countries, including Poland, Italy, Brazil, Germany, China, Portugal, Turkey and the United States. The dates of publication ranged from 2005 to 2022. Assessment of study quality using the Newcastle–Ottawa Scale yielded a median score of 6, indicating overall fair quality. No study scored below 4 points, suggesting the absence of poor-quality studies (**Table 3**).

**Table 3:** Newcastle–Ottawa scale for risk of bias & quality assessment of included studies

Author	Year	Selection				Comparability	Outcome			Total
		
		A	B	C	D	E	F	G	H	
Michalczyk et al.^27^	2022	1	1	1	1	1	1	1	1	8
Tutino et al.^28^	2022	1	1	1	1	1	1	1	1	8
Feres et al.^29^	2021	1	0	1	1	0	1	0	1	5
Grabińska et al.^30^	2021	0	1	1	1	0	1	1	1	6
Creta et al.^31^	2020	1	1	1	1	2	1	0	1	8
Anheuser et al.^32^	2018	1	1	1	1	0	1	1	1	7
Hung et al.^33^	2015	1	1	1	1	2	1	1	1	9
Li et al.^34^	2015	1	1	1	1	0	1	1	0	6
Rosa and Guerreiro^36^	2015	1	1	1	0	0	1	0	0	4
Inácio et al.^37^	2019	1	1	1	0	0	1	1	1	6
Gülşen et al.^38^	2019	1	1	1	0	0	1	1	1	6
Milanese et al.^39^	2015	0	1	1	0	0	1	1	1	5
Mindrup et al.^35^	2005	1	1	1	1	0	1	1	1	7

A: Representativeness of the exposed cohort; B: selection of non-exposed cohort; C: ascertainment of exposure; D: demonstrates outcome is not present at start; E: comparability of cohorts; F: outcome assessment; G: suitable follow up length; H: adequacy of follow-up.

Sample sizes varied considerably, ranging from 6 patients in a single-center case series to 197 in a multi-center study. In total, 322 patients were treated with hyperbaric oxygen therapy compared to 366 patients in the non-hyperbaric oxygen (control) group. The mean age was similar between groups: 57 years in the hyperbaric oxygen therapy arm versus 56 years in the control arm.

### Primary outcome

**Figure 2:**
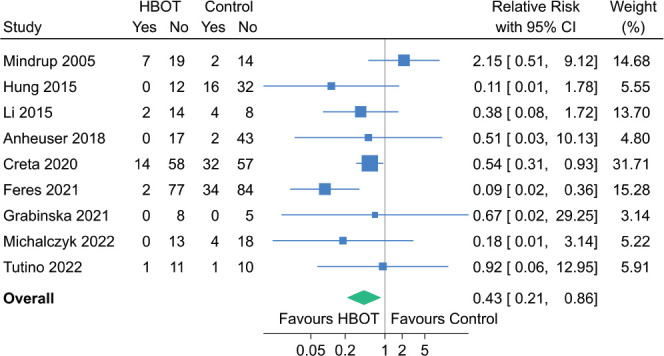
The mortality rate of Fournier’s gangrene patients in HBOT and control groups. CI: Confidence interval; HBOT: hyperbaric oxygen therapy.

The median mortality rate was significantly lower in the hyperbaric oxygen therapy group than in the control group (11.5% *vs*. 26%). Ten studies reported either a lower mortality rate in the hyperbaric oxygen therapy group or mortality rates lower than 25%. One study^30^ found no difference in mortality, with no deaths in either group. Only one study^35^ reported a higher mortality in the hyperbaric oxygen therapy group compared with the control (26.9% *vs*. 12.5%) (**Figure 2**).

### Secondary outcomes

**Figure 3:**
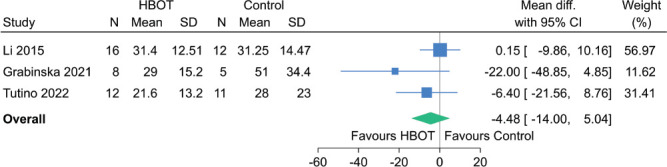
The length of stay of Fournier’s gangrene patients in HBOT and control groups. CI: Confidence interval; HBOT: hyperbaric oxygen therapy.

**Figure 4:**
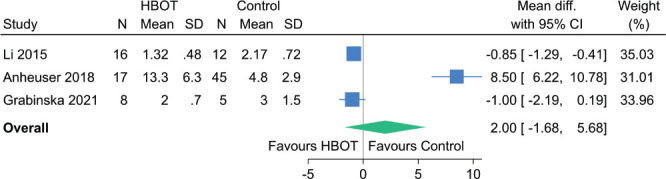
The number of debridements of Fournier’s gangrene patients in HBOT and control groups. CI: Confidence interval; HBOT: hyperbaric oxygen therapy.

Data on secondary outcomes were inconsistently reported across studies. The median hospital length of stay was slightly shorter in the hyperbaric oxygen therapy group (26 days) compared with the control group (28 days). Of the six studies reporting hospitalization time, three studies^28^,^30^,^35^ found shorter stays with hyperbaric oxygen therapy, whilst the other three^27^,^32^,^34^ reported longer stays (**Figure 3**). Regarding surgical burden, although the median number of debridements overall appeared lower in the hyperbaric oxygen therapy group (1.7 *vs*. 2.6), only four of the nine studies with control groups provided complete data on the number of debridements. Amongst these, just one study^30^ demonstrated a reduction in the number of debridements in the hyperbaric oxygen therapy arm (**Table 2** and **Figure 4**).

### Meta-analyses

Meta-analyses were performed to pool together the results from the different studies. The analysis results are summarized in **Table 4**. We observed a statistically significant difference in mortality between the two groups. Mortality was lower in the hyperbaric oxygen therapy group, with the chance of mortality only 0.43 times as large (or just over twice as low) than in the control group. The length of stay in hospital and the number of debridements did not significantly vary between groups.

**Table 4:** Meta-analysis results of primary and secondary outcomes of Fournier’s gangrene patients

	Number of studies	Heterogeneity		Treatment effect	
	
		*P*-value	*I* ^2^	Relative risk/Mean (95% CI)^a^	*P*-value
**Primary outcome**					
Mortality	9	0.16	32%	0.43 (0.21, 0.86)	0.02
**Secondary outcomes**					
Hospital length of stay	3	0.29	19%	–4.5 (–14,5.0)	0.36
Number of debridements	3	< 0.001	97%	2.0 (–1.7, 5.7)	0.29

Treatment differences for binary outcomes (primary outcome) are expressed as a relative risk, giving the risk of the outcome in the hyperbaric oxygen therapy group relative to the outcome in the control group. The treatment effects for the continuous outcome (secondary outcomes) are expressed as mean differences, showing the outcome for the hyperbaric oxygen therapy group minus the value for the control group. CI: Confidence interval.

**Figure 5:**
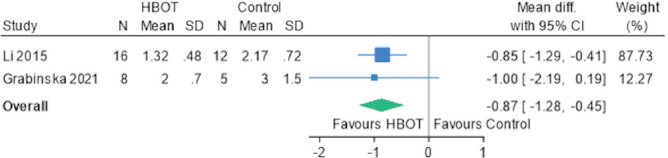
The number of debridements of Fournier’s gangrene patients after removing the outlier study The data from Anheuser et al.^33^ were omitted in sensitivity analysis. CI: Confidence interval; HBOT: hyperbaric oxygen therapy.

There was relatively little heterogeneity between studies for mortality and length of hospitalization stay, with non-significant tests of heterogeneity and relatively low *I*^2^ values (32% and 19%, respectively). However, there was a high degree of heterogeneity between studies for the number of debridements (*I*^2^ = 97%), mainly due to Anheuser’s study.^32^ Interestingly, after omitting Anheuser’s study,^32^ the number of debridements was significantly lower in the HBOT group, with a mean difference of 0.9 debridements. There was little heterogeneity between the two remaining studies (**Table 5**, and **Figure 5**).

**Table 5:** Meta-analysis results of the number of debridements after omitting the outlier study

	Number of studies	Heterogeneity		Treatment effect	
	
		*P*-value	*I* ^2^	Mean (95% CI)^a^	*P*-value
Number of debridements	2	0.82	0%	–0.9 (–1.3, –0.5)	0.29

The data from Anheuser et al.^33^ were omitted in sensitivity analysis. ^a^It was calculated as values for HOBT group minus values for control group. CI: Confidence interval; HBOT: hyperbaric oxygen therapy.

## Discussion

The primary objective of this review was to assess the impact of hyperbaric oxygen therapy on patient mortality. Our findings demonstrate a significant reduction in mortality among patients who received hyperbaric oxygen therapy compared with those who received standard care alone. The overall mortality rate in the hyperbaric oxygen group was 11.5%, compared with 26% in the control group. Meta-analysis yielded a relative risk of mortality of 0.43, indicating that patients treated with hyperbaric oxygen therapy had a 57% lower risk of death compared to controls. The control group’s mortality aligns with historically reported rates of 20–40%. Similar findings have previously been reported and published.^24^,^41^,^42^

Mindrup et al.^35^ was the only study to report an increase in mortality in the hyperbaric oxygen group. This increase in mortality may be due to selection bias, with more severely ill patients necessitating therapeutic adjuncts, i.e., hyperbaric oxygen therapy. Differences in the patient’s pre-morbid status may also explain this outlier. For instance, Feres et al.^29^ reported a 2.5% mortality in their hyperbaric oxygen group. However, the baseline characteristics of their patients differed significantly - they were younger (mean age 48 *vs*. 57), had lower rates of diabetes (36.7% *vs*. 73%) and smoked less (30.3% *vs*. 73%). Moreover, Mindrup et al.^35^ had a median follow-up of 4.2 years (range from 9 months to 10 years), significantly longer than in other studies, many of which lacked a defined follow-up period. Given the severity and complexity of Fournier’s gangrene, a longer follow-up may have allowed for more secondary events to emerge. It is well documented that delays in surgical debridement are a primary contributor to increased mortality, with rates reported up to 88%.^43^,^44^ Therefore, although the observed reduction in mortality with hyperbaric oxygen therapy is promising, it should be regarded strictly as an adjunctive therapy, considered only after urgent and thorough debridement, not as a primary intervention in the acute setting.

Our findings on secondary outcomes were more variable. Studies presented conflicting results regarding hospital length of stay, with the overall median stay only 2 days shorter in the hyperbaric oxygen group. The decision to use an adjunctive therapy may itself prolong hospitalization, potentially delaying earlier discharge to rehabilitation or community care. Furthermore, hyperbaric oxygen therapy protocols varied significantly between studies. In many cases, the protocol was either not reported^27^,^32^,^33^,^38^ or only partially reported.^28^,^37^ Given that hyperbaric oxygen therapy remains an adjunctive therapy for Fournier’s gangrene with no current international consensus on its role,^45^ such variance is unsurprising. Session durations ranged from 30 to 180 minutes, pressure levels from 2.4 to 3.0 atm, and the number of sessions from 2 to 26. The high degree of heterogeneity observed in the number of surgical debridements was primarily attributable to Anheuser’s study,^32^ which reported a substantially higher mean number of debridements compared to other included studies. While differences in institutional surgical protocols may partly explain this discrepancy, patient selection may have also contributed. In this study, the 17 patients in the hyperbaric oxygen group underwent a mean of 13.3 debridements. Notably, every patient in this group was admitted to the intensive care unit with sepsis, and 70% developed septic shock. This suggests a more severely ill patient cohort, which may explain the higher number of debridements required on average.

There was limited evidence to suggest hyperbaric oxygen therapy reduced the number of debridements required. Only Grabińska et al.^30^ reported a significant reduction in the hyperbaric oxygen group. Interestingly, 6 patients in their study presented with WBC count < 10 × 10^9^/L, and C-reactive protein < 25 mg/dL, calling into question the diagnosis of Fournier’s gangrene, or at least the severity of illness in the group compared to other studies. Several other studies^28^,^33^,^34^,^38^,^39^ utilized Fournier’s Gangrene Severity Index scoring system to describe the clinical status of the group objectively. If Fournier’s Gangrene Severity Index were uniformly applied, it would allow for more direct comparisons and may help identify patient subgroups that would benefit most from hyperbaric oxygen therapy.

To the best of our knowledge, this is one of the few systematic reviews and meta-analyses conducted on the subject of hyperbaric oxygen therapy in Fournier’s gangrene, thus addressing a significant gap in literature. It was conducted with a comprehensive search strategy, clear inclusion/exclusion criteria and followed the PRISMA guidelines to enhance transparency and reproducibility. Our bias assessment reported an overall fair quality of data. The use of meta-analytic methods, including sensitivity analyses and evaluation of heterogeneity, adds methodological strength and improves the reliability of the overall findings.

The review, however, is subject to several limitations. The inconsistency in secondary outcome reporting and the heterogeneity across studies reduce the reliability and clinical applicability of the findings. The diagnostic criteria for Fournier’s gangrene varied across studies, leading to a wide spectrum in the severity of illness among included patients. There was also a variation in study design, hyperbaric oxygen therapy protocols and outcome definitions. Not all studies reported patient characteristics by treatment group; some provided only overall data, while others omitted this information entirely, making subgroup analysis challenging. This may partly be due to all studies being retrospective, which may have led to the missing parameters. These study designs also carry a significant risk of selection bias and confounding, which could have influenced the findings. However, given the life-threatening nature of Fournier’s gangrene, prospective studies would be difficult, if not unethical, to carry out.

Finally, the cost of hyperbaric oxygen therapy is not trivial.^35^ Direct hospital costs have been reported to exceed $35,000^46^ for each course, and coverage is often lacking from insurance providers or healthcare commissioning groups. Currently in the United Kingdom, hyperbaric oxygen services are not routinely provided for any necrotizing soft tissue infections, including Fournier’s gangrene.^47^ The infrastructure required for hyperbaric oxygen service provision is costly and lacking, often necessitating patient transfers over long distances. Rosa et al.^36^ reported transfers from over 100 km away, raising logistical concerns regarding the transfer of potentially critically ill patients. The British Hyperbaric Association currently have only 10 registered hyperbaric facilities in the entire British Isles. If hyperbaric oxygen is to be more broadly recommended, further data are needed to stratify which patients are most suitable for the therapy – not only in terms of clinical outcomes, but also from a public health and cost-effectiveness perspective.

While hyperbaric oxygen therapy may offer a survival benefit in post-surgical patients, its effect on broader clinical outcomes and healthcare resource utilization remains uncertain. Ideally, high-quality randomized controlled trials with standardized hyperbaric oxygen protocols and outcome measures are needed to define its role more clearly in the management of Fournier’s gangrene; however, ethical concerns around randomizing potentially lifesaving treatment present barriers to such studies. As a result, until such studies are available, hyperbaric oxygen therapy in cases of Fournier’s gangrene should be considered on a case-by-case basis, guided by a multi-disciplinary team approach.

## Data Availability

*Not applicable.*
